# Effect of Health Shocks on Poverty Status in South Korea: Exploring the Mechanism of Medical Impoverishment

**DOI:** 10.34172/ijhpm.2021.97

**Published:** 2021-09-05

**Authors:** Chang-O Kim

**Affiliations:** ^1^Visiting Doctors Program of Medical Home, Seoul, Republic of Korea.; ^2^Institute of Social Welfare, SungKongHoe University, Seoul, Republic of Korea.

**Keywords:** Health Shock, Medical Impoverishment, Health Inequality, Sickness Benefit, South Korea

## Abstract

**Background:** South Korea has the highest out-of-pocket burden for medical expenses among the Organisation for Economic Co-operation and Development (OECD) member countries and has no formal sickness benefit system, along with United States and Switzerland, greatly increasing the risk of poverty due to a sudden illness.

**Methods:** We identify the causal effect of health shocks on poverty status and explore the mechanisms of medical impoverishment by analyzing longitudinal data from 13 670 households that participated in the representative Korean Welfare Panel Study (KOWEPS) from 2007 to 2016. In this study, we define a *health shock* as a case in which no household members were hospitalized in the previous year, but together they had more than 30 days of hospitalization in this year. The propensity score matching method was combined with a mediation analysis in this work.

**Results:** The proportion of households in absolute poverty increased by 4.6–8.0 percentage points among households that experienced a health shock compared with matched controls. The selection effects due to health shock were estimated to be 5.6–8.2 percentage points. On average, a sudden hospitalization reduces annual non-medical expenditures and equivalized disposable income by just over 3.2 million KRW (2500 USD) and 1.2 million KRW (1000 USD), respectively. Health shock induces impoverishment after one year through both the medical expense and work capacity pathways, which explain 12.8% and 12.8% of the total effect, respectively. However, when we decompose the mediation effect of a health shock on poverty status after two years, we find that a health shock leads to poverty mainly through labor force nonparticipation (9.9%).

**Conclusion:** Income stabilizing scheme to protect households that experience a health shock should be introduced as a policy alternative to confront the issue of medical impoverishment.

## Background

**Key Messages**

** Implications for policy makers**

- To be poor and sick is not only a common condition, but perhaps among the most degrading and intolerable conditions.
- Health policy experts referred to this kind of vulnerability as a ‘medical poverty trap.’
- Using South Korea as an example, our study finds empirical evidence that there is a hidden mechanism of ‘poverty → health shock → poverty.’
- Policy alternatives should therefore be enacted in both directions to alleviate health inequalities.
- Specifically, employment protection scheme to prevent labor market exit after the onset of severe illness must be considered as important alternative.

** Implications for the public**

If a sudden illness brings people falling into poverty, would the national health insurance system be sufficient? The present results indicate that a health shock, when no household members were hospitalized in the previous year but they together experienced more than 30 days of hospitalization in this year, is a cause of poverty. Thus, our findings provide additional evidence for recommending an employment protection scheme to prevent labor market exit after the onset of a severe illness. In addition, an income stabilizing scheme, such as a sickness benefit after experiencing a health shock, should be introduced as a policy alternative.

Illness and poverty commonly coexist throughout the world, causing intolerable damage to a person’s life and social stability. A severe illness can throw a household into poverty by acting as a risky life event, and, on the contrary, poverty can be a social determinant of illness through health inequality. Whitehead and her colleagues referred to this relationship as the ‘medical poverty trap.’^1^ To date, empirical evidence fully supports both directions of health inequality.^2^ In other words, it has been well established that poverty can lead to illness and illness can lead to poverty.

 However, few empirical studies have examined health inequalities in the illness → poverty direction, especially under the rubric of medical impoverishment. In the field of public health, health has been understood as the ultimate goal of welfare; thus most studies have been conducted in the poverty → illness direction.^3^ In economics, health has been conceptualized as a determinant of productivity and thus often analyzed macroscopically in the framework of new growth theory or human capital theory.^4^ Only recently have the economic consequences of illness been studied at the individual or household level.

 In Western countries, the effects of illness on poverty status have been studied primarily in the interest of developing policy to reduce early retirement among older adults.^5-10^ Zucchelli and colleagues reported that severe illness among older working individuals increases the risk of job loss by 50% to 320% based on panel data of the Household, Income and Labour Dynamics in Australia Survey.^7^ Within the tradition of labor economics, many studies have reported that illness reduces working hours and labor income and eventually induces labor market exit, despite the existence of fine employment protection legislation. Much more research has been performed in Third World countries, mainly by public health researchers examining medical impoverishment.^11-19^ For example, Xu and colleagues reported that surveys in 89 countries covering 89% of the world’s population suggest that 150 million people globally suffer financial catastrophe due to healthcare costs.^20^ Because most of the studied countries have not yet established a universal health coverage system, the main concerns of those studies were how to alleviate the economic burden of out-of-pocket medical expenses. Thus, within the tradition of public health, the more direct effects of medical expenses on poverty status have been recognized as an important research topic, but the labor market effects of illness itself have not been adequately studied.

 Although previous studies have suggested that health shocks significantly affect both economic resources and welfare at the household level, that work has serious limitations. First, little empirical evidence has addressed causality rather than an association to infer that illness produces poverty. Methodological challenges, such as a lack of panel data or measurement problems associated with endogeneity, made this kind of limitation prominent in studies conducted in Third World countries.^19-25^ Second, relatively few studies have set poverty status as an outcome variable. In the tradition of labor economics, labor supply indices, such as working hours or labor income, were frequently used as outcome variables and often analyzed at the individual level.^7-9,26-28^ However, poverty cannot be evaluated without considering issues such as household equivalization, intra-household labor substitution, and the poverty line. Third, no comprehensive analysis has addressed the mechanism of medical impoverishment from a transdisciplinary perspective. In general, studies conducted in the economics tradition have operated under the premise that illness lowers the utility of individuals by causing a loss of earning capacity,^29,30^ whereas studies conducted in the public health tradition have suggested that the phenomenon of medical impoverishment fits within the concept of catastrophic medical expenses.^12,20,31,32^ Each mechanism has sufficient theoretical evidence, but neither one provides a complete picture. To date, no empirical studies have definitively decomposed the contributions of the two separate mechanisms to explain the overall effects of health shocks.

 In this study, we evaluate the causal effects of a health shock on poverty status and identify the major mechanisms of medical impoverishment in South Korea. More specifically, we provide empirical evidence to answer the following research questions.

1. Do health shocks cause poverty?
2. If so, how do health shocks cause poverty?

###  Theoretical Mechanism: Medical Expense Versus Work Capacity Pathways

**Figure 1:**
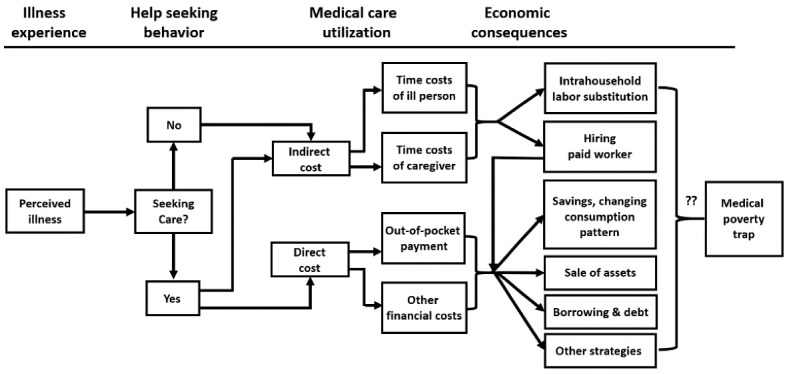
Theoretical process of medical impoverishment. Source: Author’s modification of McIntyre et al.^33^

 What is the process by which people get ill and fall into poverty? In the present study, we began with a theoretical model of medical impoverishment based on a critical review of McIntyre and his colleagues.^33^ We used the framework presented in Figure 1, widely cited by various researchers, to guide our analytic modeling.^15,34-37^ According to Figure 1, the impoverishment process is composed of 4 stages: illness experience, help seeking, medical care utilization, and economic consequences. An individual who gets sick chooses whether medical care is necessary to relieve their symptoms. However, regardless of the individual’s choice, the household will be forced to pay the associated costs, especially in the case of a new severe illness.

 The first important consideration is that the medical-care market differs significantly from the actual competitive market model under certainty. Because medical-care products are acknowledged as complementary to risk-bearing, they are almost always purchased at the household level, even if the product does not promise to restore a household member’s condition.^32,38^ Uncertainty as to the quality of the product is more intense than with any other important commodity.^39^ Furthermore, pricing practices in the medical industry depart sharply from the competitive norm. Because of a collective monopoly of physicians, households cannot avoid following the price policy of hospitals. This process is similar to paying taxes. Assuming that total household consumption (TC) is fixed, overspending for a direct cost (ie, medical treatment and related financial costs, DC) will always lead to the risk of deprivation (ie, poverty) because non-medical consumption (NC) will always decrease to balance the level of total consumption (TC = DC + NC).

 Second, even if individuals decide not to use a medical care service at all, a new severe illness will reduce the healthy time (HT) of household members. According to Grossman’s health capital model,^40^ ill health reduces HT and induces time lost (ie, is an indirect cost, IC) from market and nonmarket activities because total time (Ω = 365 days) is constant for everyone (Ω = HT + IC). Assuming no income stabilizing scheme (eg, sickness benefit) is operational, time lost in labor market activity will decrease labor income, which increases the risk of poverty by removing economic resources. Intra-household labor substitution could minimize the effects of the indirect cost.^41^ For example, other household members could decide to participate in labor market activity to compensate for the reduction in labor income caused by illness. In the real world, however, it is acknowledged that a health shock has spillover effects on the employment and incomes of non-sick household members. According to an empirical analysis in the Netherlands, household income falls by 50% more than the income loss experienced by the person admitted to a hospital.^9^ This is probably because an adult who was initially working has an increased probability of leaving employment when their spouse gets a severe illness.

###  Healthcare and Labor Market in Korea

 Although Figure 1 was drawn based on qualitative studies in low- and middle-income countries, it seems suitable to South Korea (hereafter Korea) as well. Korea is economically prospering and is 12th in the World Bank’s most recent gross domestic product country ranking. Furthermore, Korea has had a national health insurance system that offers universal coverage since 1989. However, the health insurance system does not seem to sufficiently protect Korean households from overspending on medical expenses.^42^ In a comparative study, the out-of-pocket payment share of total household healthcare consumption in Korea was found to be 4.7%, which is the highest among the 34 countries in the Organisation for Economic Co-operation and Development (OECD average 2.8%).^43^ The percentage of Korean households facing catastrophic medical expenses was 2.6%, which is similar to countries that have no national health insurance system, such as China (2.8%) or Vietnam (2.9%).^12^ This reflects the extensive use of copayments, non-coverage for many treatments, and only partial coverage for expensive inpatient care provided by the Korean health insurance system.^12,44^ Thus, overspending on direct medical costs could be the first pathway to medical impoverishment in Korea.

 At the same time, Korea is one of the 34 OECD countries, along with the United States and Switzerland, that has no formal sickness benefit system. A sickness benefit is commonly defined as cash benefits that replace the wages of workers who miss work due to illness.^45^ It is understood as a key component of social security systems in Western societies and was introduced at an early stage in the development of welfare capitalism.^46,47^ In Germany, for example, the sickness benefit was implemented at the end of the 19th century, and 100% of wages are paid for up to 6 months of sick leave. The Netherlands has 3 major cash transfer programs for individuals of working age who do not work: disability insurance, unemployment insurance, and social assistance.^9^ Through strong employment protection legislation and collective bargaining by unions, workers in the Netherlands can receive 70% to 100% of their net salary for at least 6 years after they leave the workplace due to severe illness.

 It is uncertain which pathway of medical impoverishment is more important in Korea. However, considering the characteristics of the dual labor market in Korea, overspending on indirect costs must also be an influential pathway that leads households into poverty. The proportion of precarious workers in the labor market in Korea is 32%, and 92.6% of them are classified as temporary or atypical workers with poor working conditions.^48^ In addition, the unionization rate in Korea is 10%, far below the OECD average of 32%.^49^ Thus, although it has not yet been empirically verified, a hidden pathway of precarious work → health shock → labor force nonparticipation → poverty in Korea seems inevitable.

## Methods

###  Data Source and Study Subjects

 Our analysis uses data from ten waves (2007–2016) of the Korean Welfare Panel Study (KOWEPS). The KOWEPS is an annual longitudinal panel survey of Korean citizens over the age of 15 years that has been conducted since 2006. It includes 18 856 individuals from 7072 households who were recruited by a two-stage stratified cluster sampling at baseline. The KOWEPS contains detailed information on health status, labor market activities, household income, and expenditures. In addition, since about 50% of the total sample is selected from a low-income group (less than 60% of the national median income at the time of sampling), the data are especially appropriate for low-income-targeted policies and poverty research.

 In the actual analysis, using the methodology of Jenkins and Riggs,^50^ we considered the following subsamples:

 (a) The subset of households at risk of a health shock from 2010 to 2012 that experienced a health shock from 2010 to 2012.

 (b) The subset of households at risk of a health shock from 2010 to 2012 that did not experience a health shock from 2010 to 2012.

 Samples a and b contain 8 consecutive years of longitudinal survey data; we used those data to examine the long-term effects of a health shock (t = 2010, 2011, 2012) on selection for 3 years (from t-3 to t-1) and impoverishment for 4 years (from t+1 to t+4). We constructed and pooled a total of 6 subsamples into the final sample of the study (Supplementary file 1, Table S1). Among the households missing information for none of the main variables, 398 households experienced a health shock in 2010–2012 (sample a), and 13 272 households that were at risk of a health shock did not experience one in 2010–2012 (sample b). Because we used sample a (398 exclusive households) and propensity score–matched subjects (398 non-exclusive households) from sample b (13 272 non-exclusive households) for most of the analyses, the standard weight of the KOWEPS was rarely applied in this study.

###  Independent Variables

 The following list summarizes the conceptual definitions of *health shock* used in the major precedent studies:

1. Sudden deterioration in a person’s health^6^
2. Large unexpected major illness^51^
3. New severe health event^52^
4. Illness or health event that changes the ability to perform activities of daily living to an uncertain extent^53^
5. Unscheduled hospitalization.^9^

In most of the literature, health shocks have been described using the concepts of volatility and uncertainty, indicating that summarizing an individual’s health using a static overall average is insufficient and that health shocks must account for intrapersonal intertemporal heterogeneity. John H. Cochrane is a representative scholar who points out that unpredictability is an important factor in health.^54^ After a pioneering review of various exogenous variables that significantly affect consumption smoothing, he concluded that an unexpected major illness (loss of more than 100 days of work due to illness) was an event that could properly be called an *idiosyncratic shock*.^54,55^ Since then, the term *health shock* has been widely used in economics and public health studies.^6,9,51-53^ A health shock is not a simple change in health status, but rather an event that causes a ‘reduced capability’ and prevents an individual from converting a given resource into utility.^56^ Thus, to be called a health shock, a health event must be severe enough to induce deterioration in the ability to maintain living standards.^51^

 Health shocks have been operationalized in a variety of ways. In practice, the measures can be divided into 8 categories^30^: (1) self-reported health status; (2) health limitation on ability to work; (3) activities of daily living; (4) nutritional status; (5) expected or future mortality; (6) clinical assessments of mental health; (7) the presence of chronic and acute conditions; (8) the utilization of medical care. In this study, we define health shocks using the length of a hospital stay.^9,57-60^ Basically, it reflects both the severity of an illness and health limitations on the ability to work.^8^ As a health shock indicator, the volatility of inpatient days (*V*_it_) is measured using the following equation:


$$V_{it}=I_{it}-I_{i,t-1}$$

where *I*_it_ denotes total inpatient days for all household members each year. By using this method, we can use the health shock variable to account for the uncertainty of illness. We applied a threshold to make simple indicator.^32^ The threshold approach is necessary because a health shock can be reasonably defined only if the event is severe enough to reduce earning capability. A health shock (*S*_it_) can be measured by the following equation:


$$S_{it}=\left\{\begin{array}{l} 1\;if\;V_{it}\geq d\\ 0\;if\;V_{it}<d \end{array}\right.$$

where *d* denotes the specified threshold, which represents the point at which a change in the length of hospital stay is considered to impose a severe disruption on work capacity or living standards (0 < *d* ≤ 365). This is obviously a matter of judgment.^32^ In this study, we set *d* to 30. Thus, a health shock is defined as an event in which the length of hospitalization increased by more than a month over the previous year.

**Figure 2:**
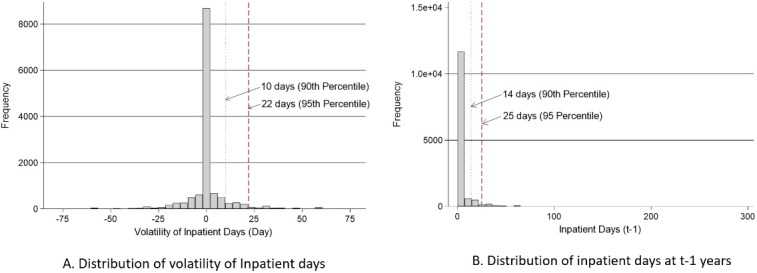
Distribution of the Volatility of Inpatient Days and Actual Days of Hospitalization Within the Study Population (N = 13 670). Source: Korean Welfare Panel Study, 2007–2016.

Figure 2A shows the distribution of *V*_it_ among the 13 670 eligible households from KOWEPS (t = 2010, 2011, 2012): 61.1% of total households recorded zero (*V*_it_ = 0); 20.6% reported spending at least one extra day in the hospital compared with the preceding year (*V*_it_ > 0), and 18.3% reported spending at least one fewer day than the previous year (*V*_it_ < 0). The proportion of households facing more than 10 and 22 days of hospital-stay increase over the preceding year were 10% and 5%, respectively. Figure 2B shows the distribution of actual days of hospitalization in year t-1. As might be expected, 75.6% of total households did not experience any hospitalization for any household members (median value 0). The proportion of households facing fewer than 14 days, from 14 to 29 days, and more than 30 days of admission were 14.1%, 7.0%, and 3.4%, respectively.

 Based on that descriptive analysis, we simplified our operational definition of a health shock. We replaced *I*_i,t–1 _(observed value of households) with zero (median value of population sample). Therefore, in this study, a health shock satisfies the two following conditions:

1. More than 30 days of hospitalization in year t
2. No hospitalization in year t-1.

Here, *days of hospitalization* is the sum of all hospital stays by any household member. In this study, we did not restrict the type of illness, purpose of hospitalization, or type of medical institution.

###  Dependent Variables

 The outcome variable in this study is poverty status, which we define as ‘unacceptable deprivation,’ when household non-medical expenditures fall below the level needed for a household to meet the basic needs of living.^61^ Every year from 1999 to 2015, the Korean government measured the minimum cost of living according to household size by the market basket method and announced it as the official poverty line for operating the public assistance program. Over the past 10 years, the absolute poverty rate (based on household expenditure) of the general Korean population has increased from 6.5% in 2007 to 9.0% in 2016. A full basket contains 11 kinds of necessities, including medical care. Because medical care generally costs as much as 4.4% of household income almost every year, we used 95.6% of the minimum cost of living as the absolute poverty line in this study. Poverty status was measured for 8 consecutive years, from 3 years before to 4 years after the onset of a health shock, to examine whether selection or impoverishment effects occurred. In this study, the impoverishment effect is defined as a difference in the poverty rate between households that experienced a health shock (sample a) and matched controls that did not during the period beginning 1 year after the onset of a health shock (ie, from t+1 to t+4). The selection effect is defined as a difference in the poverty rate between the non–health shock group (sample b) and the non–health shock matched controls during the period before the onset of a health shock (ie, from t-3 to t-1).

###  Mediator Variables

 Previous studies measured overspending on direct costs using the financially catastrophic or high-cost method.^31,62,63^ In this study, we define overspending on direct costs, taken as the first mediator variable, as a catastrophic medical expense that exceeds 10% of total household income. Overspending on indirect costs can be measured in various ways, such as labor income, working hours, number of working household members and labor force participation.^29,30^ In this study, we define overspending on indirect costs, taken as the second mediator variable, as labor force nonparticipation, ie, no household members participate in labor market activities. These variables were measured at year t+1 to show the temporal relationship between the independent (year t) and outcome variables (year t+1, t+2).

###  Statistical Analyses

 Because health shocks do not occur randomly, potential confounding and selection biases should be accounted for by a statistical technique, such as the propensity score method. The idea of propensity score matching is to perform 1:1 matching of subjects who have the same (or nearest) probability of experiencing a health shock. Thus, even if the actual occurrence of health shocks differs between the two groups, the distribution of conditioning variables will be the same.^64^ In other words, if the propensity score could be estimated using all the observed covariates associated with selection bias, we could theoretically infer the relationship between health shocks and poverty as causality.

 Multivariate logistic regression analyses were used to estimate the predicted probability of 36 variables, including (1) predictors of health shock occurrence, (2) confounding determinants of poverty, and (3) demographic characteristics (Supplementary file 1, Table S2). We included variables that define poverty status by measuring it at years t-3 and t-2 as lagged dependent variables. Because the analytic data are pooled from 3 subsamples based on the year of health shock occurrence (t = 2010, t = 2011, t = 2012), the propensity score estimations were performed from the same 3 subsamples (n = 4813, n = 4553, n = 4304). Nearest neighborhood matching without replacement was used within a caliper of 0.25σ_p_ (standard deviation of the propensity score) with the user-developed *psmath2 *program in Stata 16.0 (StataCorp, College Station, TX, USA).

 To identify the mechanism of medical impoverishment, we used the causal mediation analysis proposed by Coffman.^65^ In general, mediation is tested using regression analyses, as suggested by Baron and Kenny.^66^ However, as several authors have pointed out, this classic approach cannot satisfy the conditional independent assumption between the mediator (M) and the dependent variable (Y) if causal inferences are to be made about the M → Y relationship.^67,68^ An alternative of Coffman^65^ is to use the propensity score method in the M → Y estimation. Here, the propensity score is the probability that a household experiences a particular event of M (ie, overspending direct or indirect cost at year t+1). The rationale underlying the propensity score approach is that incorporating a model for mediator selection will produce an unbiased estimate of the effect of M on Y. Estimating propensity score of M by using all the observed covariates and incorporating the results into the classic regression model would produce an improvement over the current classical approach. Another option is to include all confounders in the classic regression model directly; although this often leads to multicollinearity. An advantage of propensity scores is that they reduce the large number of potential confounders into single numerical summary.

**Figure 3:**
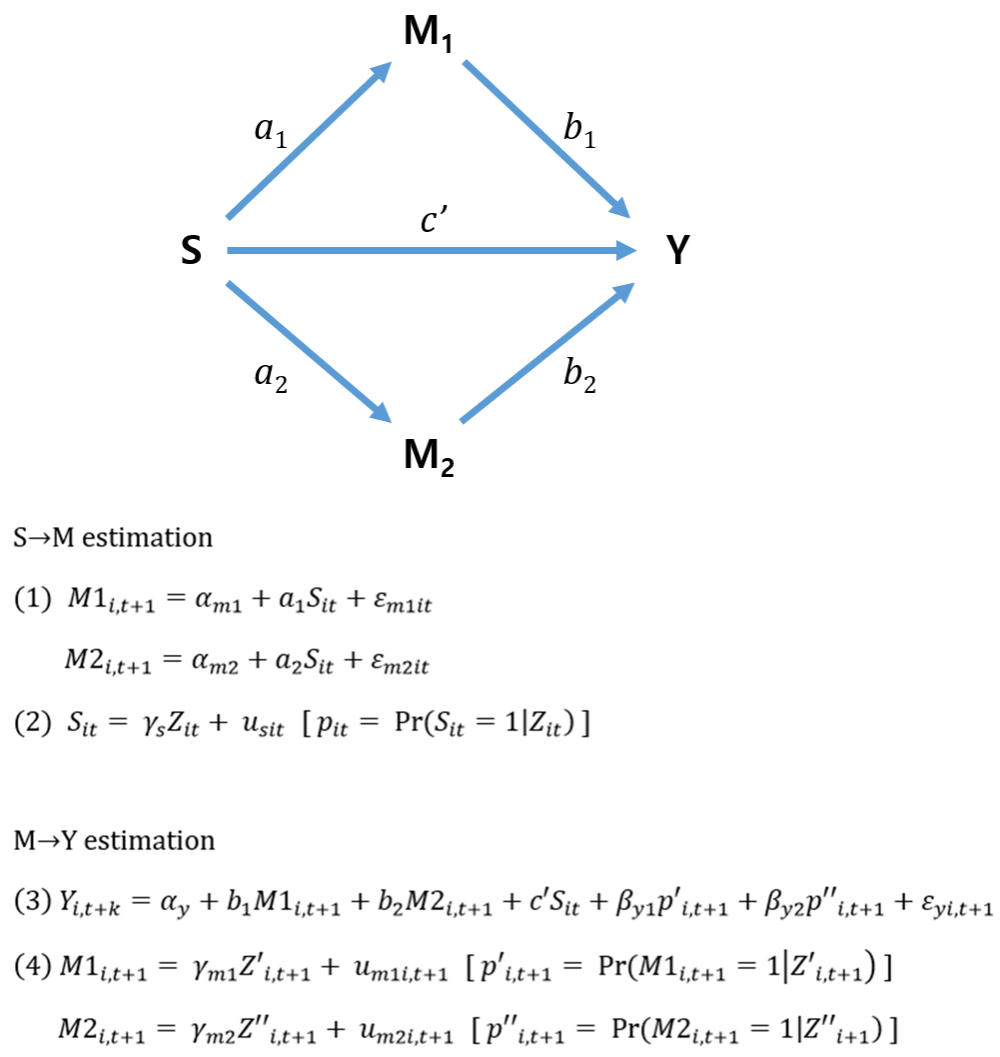
Structural Equations to Perform the Causal Mediation Analysis. In this model, the total effect (c) of a health shock (S) on poverty status (Y) through mediators (M1, M2) is decomposed as follows: c = a_1 _b_1_ + a_2 _b_2_ + c’. Here, a_1_, a_2_, b_1_, b_2_ and c’ can be estimated through the structural equations above.

Figure 3 shows the structural equations that follow the proposition of Coffman^65^ for making a causal inference in a mediation analysis. First, the S → M estimation process is similar to the propensity score matching method already described above. Because we used matched samples based on the propensity score of a health shock occurrence [*pit = *P_r_ (*S*_it_*=*1|Z_it_)], the causal effect of S on M1 and M2 (ie, *a*_1_, *a*_2_) can be estimated by a linear probability model (equation 1). Second, the M → Y estimation process can be performed through an ordinary least squares (OLS) regression model with the same matched sample used to estimate the S → M process. However, to estimate the unbiased effects of S, M1, and M2 on Y (ie, *c’*,* b*, *b*_2_), additional variables should be incorporated into equation 3. In this study, we propose new structural equations to estimate the conditional probability that a catastrophic medical expense $\left[{p^{\prime }}_{i,t+1}=\mathrm{Pr}\left(M1_{i,t+1}=1\left|{Z^{\prime }}_{i,t+1}\right.\right)\right]$ or labor market nonparticipation $\left[{p^{\prime \prime }}_{\;i,t+1}=\mathrm{Pr}\left(M2_{i,t+1}=1\left|{Z^{\prime \prime }}_{\;i,t+1}\right.\right)\right]$ will occur in year t+1 (equation 4). Because the newly developed propensity scores (ie, *p’*, *p’’*) are one-dimensional and contain information about mediator selection, we can estimate the causal effect of M → Y by putting those variables into the regression model in equation 3.^65^ The detailed process used to estimate the propensity scores of each mediator are described in Table S3. Lagged dependent variables measured at years t-3, t-2, and t-1 are included in the list of conditional variables.

 In this way, the causal mediation effect of a health shock on impoverishment can be calculated as follows. We can also decompose the proportion that each pathway contributes to the total effect of impoverishment.

 Total impoverishment effect: *c*

 Direct effect of health shock: *c’*

 Mediation effect of direct costs (medical expense pathway): *a*_1_*b*_1_

 Mediation effect of indirect costs (work capacity pathway): *a*_2_*b*_2_

 Mediators’ selection effect: $c-c^{\prime }-a_{1}b_{1}-a_{2}b_{2}$

$\left(\frac{c^{\prime }}{c}\right)+\left(\frac{a_{1}b_{1}}{c}\right)+\left(\frac{a_{2}b_{2}}{c}\right)+\left(\frac{c-c^{\prime }-a_{1}b_{1}-a_{2}b_{2}}{c}\right)=1$

## Results

###  Characteristics of Study Subjects

 Among the 13 670 households eligible for analysis, 398 (2.9%) households experienced a health shock from 2010 to 2012. The baseline characteristics according to group assignment and the results of the 1:1 matching are summarized in Table 1. Households with a health shock were older, more likely to have low education, and had a smaller family size than the non-matched control group. The average value of inpatient days in year t was 62 (standard deviation 44.3 days) in the health-shock group. The right column of Table 1 shows the baseline characteristics of the propensity-matched group. Unlike the non-matched control group in the left column, the propensity matched subjects were well balanced between groups according to the independent variables listed in Table 1 (*P*:.187–.832). The average number of inpatient days for the matched control group in year t was 5.3 days (standard deviation 20.8 days).

**Table 1:** Baseline Characteristics of Study Subjects

	**Entire sample (N = 13 670)**			**Propensity-Score Matched Sample (n = 796)**		
	**Health Shock Group** **(n = 398)**	**Non-matched Control (n = 13 272)**	***P***	**Health Shock Group (n = 398)**	**Matched Control** **(n = 398)**	***P***
Health shock occurrence						
2010	32.2	35.3	.196	32.2	32.2	1.000
2011	38.4	33.2	.027	38.4	38.4	1.000
2012	29.4	31.6	.363	29.4	29.4	1.000
General characteristics (t year)						
Number of family members	2.3 ± 1.2	2.6 ± 1.3	<.001	2.3 ± 1.2	2.4 ± 1.2	.769
Living with children under 20	18.1	33.3	<.001	18.1	20.1	.471
Living with parents over 65	67.6	48.4	<.001	67.6	65.6	.548
Living in urban area	65.1	74.6	<.001	65.1	65.8	.823
Beneficiary of medical aid	12.1	8.4	.010	12.1	12.8	.747
Arrearage of health insurance premium	12.3	8.6	.010	12.3	13.3	.671
Age of householder	65.0 ± 13.0	58.6 ± 15.0	<.001	65.0 ± 13.0	63.9 ± 13.2	.219
Age^2^ of householder	4397 ± 1615	3657 ± 1735	<.001	4397 ± 1615	4255 ± 1612	.215
Female householder	31.4	27.4	.079	31.4	35.2	.259
Householder’s educational year 10-11	23.4	27.4	.076	23.4	25.9	.441
Householder’s educational year ≥12	7.5	23.6	<.001	7.5	8.0	.791
Divorced or separated	35.2	30.4	.043	35.2	39.7	.187
Unmarried	2.8	4.4	.114	2.8	3.0	.832
Current health characteristics (t year)						
Inpatient days	62.0 ± 44.3	4.1 ± 17.0	<.001	62.0 ± 44.3	5.3 ± 20.8	<.001
Subjective health	2.8 ± 0.9	3.4 ± 0.9	<.001	2.8 ± 0.9	3.2 ± 0.9	<.001
No. of households of chronic illness	1.4 ± 0.8	1.0 ± 0.8	<.001	1.4 ± 0.8	1.2 ± 0.8	.001
No. of households of disability	0.4 ± 0.6	0.2 ± 0.5	<.001	0.4 ± 0.6	0.3 ± 0.5	.091
No. of households of smoking	32.9	36.9	.107	32.9	36.9	.234
No. of households of alcohol abuse	8.5	11.2	.095	8.5	9.1	.802
Forgone medical care	2.3	1.5	.194	2.3	1.8	.613
No. of private medical insurance	0.55 ± 0.83	0.80 ± 0.97	<.001	0.55 ± 0.83	0.61 ± 0.86	.365
Private medical insurance premium	103 ± 179	158 ± 238	<.001	103 ± 178	114 ± 191	.499
Past health characteristics (t-3 year)						
Inpatient days	10.7 ± 30.2	7.3 ± 28.8	<.001	10.7 ± 30.2	9.3 ± 31.1	.220
Subjective health	3.1 ± 0.9	3.4 ± 0.9	<.001	3.1 ± 0.9	3.1 ± 1.0	.544
No. of households of chronic illness	1.2 ± 0.8	1.0 ± 0.9	<.001	1.2 ± 0.8	1.2 ± 0.9	.111
No. of households of disability	0.3 ± 0.6	0.2 ± 0.5	.011	0.3 ± 0.6	0.3 ± 0.5	.734
No. of households of smoking	41.7	40.5	.628	41.7	43.2	.667
No. of households of alcohol abuse	17.6	15.7	.317	17.6	17.1	.851
Forgone medical care	3.0	2.3	.326	3.0	4.5	.264
No. of private medical insurance	0.48 ± 0.76	0.70 ± 0.67	<.001	0.48 ± 0.76	0.50 ± 0.73	.528
Private medical insurance premium	90 ± 180	122 ± 199	<.001	90 ± 181	101 ± 214	.343

Notes: Proportion (%). Mean ± standard deviation. *P* values are calculated between group values from chi-square test and independent *t* test according to the variable characteristics. Source: Korean Welfare Panel Study, 2007-2016.

###  Effect of Health Shocks on Impoverishment

**Figure 4:**
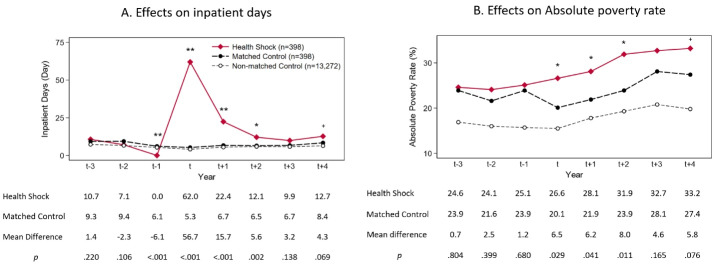
Changes in the Mean Values of Inpatient Days and the Absolute Poverty Rate Before and After a Health Shock. *P *values were calculated between the health shock and matched control groups by the Kruskal-Wallis or chi-square test (^+^*P* <.010, * P <.050, ** P <.001). Source: Korean Welfare Panel Study, 2007–2016.

 Selection and impoverishment effects were examined by tracing poverty status from 3 years before health-shock onset until 4 years after health-shock onset. As shown in Figure 4A, total inpatient days in the health shock group increased substantially between years t and t+2 (*P* ≤ .002), and then the differences between groups disappeared after year t+3 (*P* :.069–.138). This suggests that our definition of health shock well reflects the uncertainty of illness.

Figure 4B shows the changes in poverty status for 3 groups — health shock (◆), matched control (●), and non-matched control (○). In the health-shock group, the proportion of households living below the absolute poverty line increased over the 8-year span by 8.6 percentage points (from 24.6% to 33.2%). By contrast, among those at risk of a health shock who did not experience one, the proportion of absolute poverty increased only 3.5 percentage points (from 23.9% to 27.4%) in the matched control group and 2.9 percentage points (from 16.9% to 19.8%) in the non-matched control group. From year t-3 to year t-1, the poverty status between the health-shock and matched control groups did not differ significantly. For example, at the t-3, t-2 and t-1 time points, the differences in the poverty rate between the matched groups were 0.7 (=24.6–23.9, *P*= .804), 2.5 (=24.1–21.6, *P*= .399) and 1.2 (=25.1–23.9, *P*= .680) percentage points, respectively. This implies that selection bias due to health-shock occurrence was successfully controlled through the propensity score matching method. The selection effects of a health shock (ie, difference in poverty rate between the matched and non-matched control groups from years t-3 to t-1) were estimated to be 5.6–8.2 percentage points.

**Figure 5:**
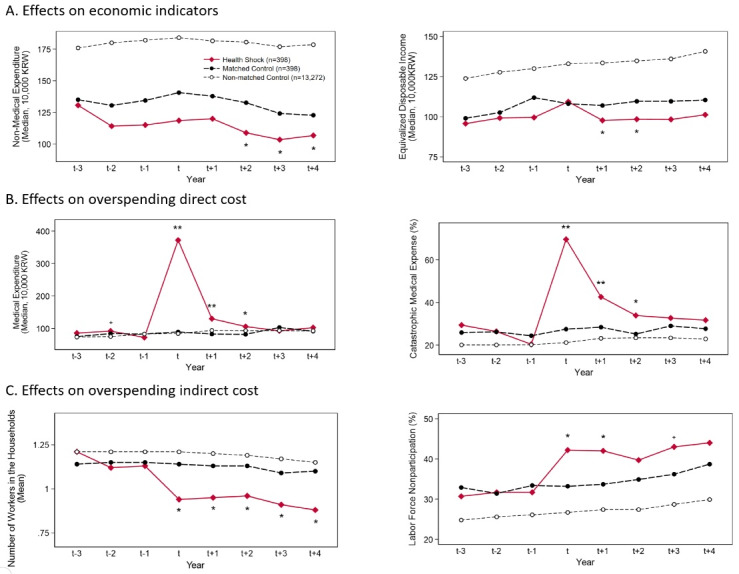
Changes in Variables Associated With Medical Impoverishment Before and After a Health Shock. *P *values were calculated between the health shock and matched control groups by the Kruskal-Wallis or chi-square test (^+^*P* <.010, *P <.050, **P <.001; 10 000 KRW = 8.2 USD). Source: Korean Welfare Panel Study, 2007–2016.

 From year t+1 to year t+4, difference in trend of poverty rate was observed between the health-shock and matched control groups. For example, at the t+1, t+2, t+3 and t+4 time points, the differences in the poverty rate between those two groups were 6.2 (= 28.1–21.9, *P*= .041), 8.0 (=31.9–23.9, *P*= .011), 4.6 (=32.7–28.1, *P*= .165), and 5.8 (=33.2–27.4, *P*= .076) percentage points, respectively. In other words, the impoverishment effect of a health shock was 4.6–8.0 percentage points, and no subsequent recovery occurred for up to 4 years after the onset of a health shock. Figure 5A shows the effects of a health shock on the other economic indicators associated with poverty. Similar results were obtained when the outcome variables were replaced with continuous variables such as household non-medical expenditures and equivalized disposable income.

###  Results of Sensitivity Analysis

Table 2 provides results of sensitivity analysis to verify the robustness of definition of health shock. When we modified the definition of health shock as an event resulting in more than 30 days of hospitalization in year t, regardless of inpatient day in year t-1, the difference in poverty rate between health shock and matched control groups was not significant (*P*= .687-.741; Table 2-A). In contrast, when the health shock group was selected from among households who had not been hospitalized in the previous year, the impoverishment effect was significant as the threshold approached 30 days (*P*= .308-.787; Table 2-B, *P*= .096-.263; Table 2-C). This implies that not only the number of inpatient days, but also the volatility of these days are main contributors to the rise in poverty rate.

**Table 2:** Sensitivity Results to Verify the Effects of Health Shocks on Changes in Absolute Poverty Rate Using Alternative Definitions and/or Conditions

	**n**	**t-3**	**t-2**	**t-1**	**t**	**t+1**	**t+2**	**t+3**	**t+4**
A. Using alternative definition (*I*_it_ ≥ 30)									
Health shock	786	24.4	24.6	26.2	24.8	25.6	27.7	29.5	29.8
Matched control	786	22.5	24.2	24.1	23.2	26.5	28.6	30.3	30.7
*P*		.372	.860	.323	.443	.687	.695	.741	.701
B. Using alternative threshold (*I*_it_ ≥ 14, *I*_i,t–1_ = 0)									
Health shock	867	22.5	21.6	21.1	24.0	24.8	27.6	28.7	27.2
Matched control	867	22.4	21.6	19.5	20.4	23.9	24.7	26.5	26.6
*P*		.954	1.000	.403	.073	.654	.172	.308	.787
C. Using alternative threshold (*I*_it_ ≥ 20, *I*_i,t–1_ = 0)									
Health shock	609	23.0	22.5	22.2	24.6	26.8	29.9	31.2	29.9
Matched control	609	21.5	21.0	20.2	19.4	24.0	26.3	27.1	25.6
*P*		.535	.532	.400	.027	.263	.161	.115	.096
D. Using mutually exclusive sample (*I*_it_ ≥ 30, *I*_i,t–1_ = 0)									
Health shock	398	24.6	24.1	25.1	26.6	28.1	31.9	32.7	33.2
Matched control	398	23.6	21.6	23.9	20.1	21.6	23.4	27.4	26.9
*P*		.740	.399	.680	.029	.033	.007	.104	.053
E. Using alternative wave (t = 2012, 2013, 2014)									
Health shock	453	23.6	22.3	28.0	30.7	29.7	27.6	28.3	27.4
Matched control	453	19.9	20.3	25.2	24.7	26.2	20.6	20.0	18.0
*P*		.171	.465	.328	.045	.243	.016	.038	.086

Note: *I*_it_, total inpatient days for all household members in year t. Notes: The matched control group was resampled according to definition of health shock using propensity matching. Details such as regression model, conditional variables, matching program, and caliper size were the same as described in the Methods section. Source: Korean Welfare Panel Study, 2007-2016.

 The fourth and fifth panels show the results when the same criteria for health shock were applied to the alternative samples. In the original sample, a total of 18 households were resampled during the process of matching at different periods. After constructing a mutually exclusive sample, by replacing these duplicate cases, the impoverishment effect was unchanged compared to the original sample (*P*= .007-.104; Table 2-D). Even after the period of health shock occurrence was reset from 2012 to 2014 (ie, instead of 2010 to 2012), the main results of this study did not change (*P*= .038-.243; Table 2-E).

###  Effects of Health Shocks on Medical Expenses and Work Capacity

Figure 5 also shows changes in the direct and indirect costs of a health shock. According to Figure 5B, the proportion of households who paid for a catastrophic medical expense increased significantly from years t to t+2 in the health shock group (*P* ≤ .007) but recovered to a non-significant level by year t+3 (*P*= .223–.496). By contrast, in households that experienced a health shock, the number of household members who participated in the labor market dropped significantly and did not recover to the level before the onset of the health shock (*P* ≤ .022; Figure 5C). Also, the labor force nonparticipation rate in the health-shock group (Figure 5C) increased over the four years after the onset of the health shock (*P* ≤ .164). This suggests that the direct and indirect costs of a health shock can produce temporary and chronic poverty, respectively, through different mechanisms.

###  Mediation Effect of Medical Impoverishment

Table 3 shows the results from the OLS regression model used to estimate the coefficients of a health shock on overspending on direct and indirect costs in year t+1 (S → M estimation). A health shock increased the probability of a catastrophic medical expense and the labor force nonparticipation rate in next year by 14.2 (β = 0.142, SE = 0.034, *P* < .001) and 8.3 percentage points (β = 0.083, SE = 0.034, *P* = .016), respectively.

**Table 3:** OLS Regression Model to Evaluate the Effects of a Health Shock on Catastrophic Medical Expenses and Labor Force Nonparticipation After 1 Year (S → M Estimation, n = 796)

	**Catastrophic Medical Expense (t+1)**		**Labor Force Nonparticipation (t+1)**	
	**β (SE)**	***P***	**β (SE)**	***P***
Health shock (t)	0.142 (0.034)	<.001	0.083 (0.034)	.016

Abbreviations: OLS, ordinary least squares; S, independent variable; M, mediator variable; β, beta coefficient; SE, standard error. Source: Korean Welfare Panel Study, 2007-2016.

Table 4 shows whether the coefficients of a health shock on poverty status in year t+1 changed when the mediator variables measured in year t+1 were incorporated into the regression model (M → Y estimation). The first panel shows the results of the unadjusted model: a health shock increased the probability of impoverishment after one year by 6.3 percentage points at a significance level of 0.05 (β = 0.063, SE = 0.031, *P* = .041). However, when we added mediators to the unadjusted model, the effect of a health shock on impoverishment was attenuated to the level of statistical non-significance (β = 0.025, SE = 0.029, *P* = .395). The results were essentially unchanged even after incorporating propensity scores for each mediator into the original regression model (β = 0.043, SE = 0.028, *P* = .135). According to the results from our causal mediation model, both a catastrophic medical expense (β = 0.057, SE = 0.032, *P* = .081) and labor force nonparticipation (β = 0.097, SE = 0.044, *P* = .030) were more important factors than a health shock in producing medical impoverishment in year t+1.

**Table 4:** OLS Regression Analyses to Evaluate the Effects of a Health Shock (t), Catastrophic Medical Expense (t+1), and Labor Force Nonparticipation (t+1) on Poverty Status in Year t+1 (M → Y Estimation, n = 796)

	**Unadjusted Model**		**Classic Mediation Model**		**Causal Mediation Model**	
	**β (SE)**	***P***	**β (SE)**	***P***	**β (SE)**	***P***
Health shock (t)	0.063 (0.031)	.041	0.025 (0.029)	.395	0.043 (0.028)	.135
Catastrophic medical expense (t+1)			0.120 (0.031)	<.001	0.057 (0.032)	.081
Labor force nonparticipation (t+1)			0.258 (0.031)	<.001	0.097 (0.044)	.030
Propensity score 1					0.523 (0.094)	<.001
Propensity score 2					0.176 (0.063)	.005

Abbreviations: OLS, ordinary least squares; M, mediator variable; Y, outcome variable; β, beta coefficient; SE, standard error. Notes: A propensity score 1 is the conditional probability of a catastrophic medical expense in year t+1 and is estimated using the logit model reported in Table S3. A propensity score 2 is the conditional probability of labor force nonparticipation in year t+1 and is also estimated using the logit model reported in Table S3.

Table 5 shows the results of the causal mediation analysis to determine the delayed effect of a health shock on impoverishment after two years. The first panel shows the results of the unadjusted model, which indicate that a health shock increases the probability of impoverishment after two years by 8.0 percentage points (β = 0.080, SE = 0.032, *P* = .011). When we added mediators and the propensity scores of each mediator to the unadjusted model, the effect of a health shock on impoverishment in year t+2 was reduced from 0.080 (unadjusted model) to 0.067 (causal mediation model). However, statistical significance was maintained at the significance level of 0.05 (β = 0.067, SE = 0.029, *P* = .023). Thus, overspending on direct and indirect costs operate not as complete mediators but as partial mediators. Labor force nonparticipation remained a significant mediator explaining impoverishment two years after the occurrence of a health shock (β = 0.095, SE = 0.045, *P* = .038). In contrast, catastrophic medical expense was not a significant mediator at that time point (β = 0.002, SE = 0.033, *P* = .958).

**Table 5:** OLS Regression Analyses to Evaluate the Effects of a Health Shock (t), Catastrophic Medical Expense (t+1), and Labor Force Nonparticipation (t+1) on Poverty Status in Year t+2 (M → Y Estimation, n = 796)

	**Unadjusted Model**		**Classic Mediation Model**		**Causal Mediation Model**	
	**β (SE)**	***P***	**β (SE)**	***P***	**β (SE)**	***P***
Health shock (t)	0.080 (0.032)	.011	0.046 (0.030)	.128	0.067 (0.029)	.023
Catastrophic medical expense (t+1)			0.085 (0.033)	.009	0.002 (0.033)	.958
Labor force nonparticipation (t+1)			0.270 (0.032)	<.001	0.095 (0.045)	.038
Propensity score 1					0.688 (0.096)	<.001
Propensity score 2					0.168 (0.064)	.009

Abbreviations: OLS, ordinary least squares; M, mediator variable; Y, outcome variable; β, beta coefficient; SE, standard error. Notes: A propensity score 1 is the conditional probability of a catastrophic medical expense in year t+1 and is estimated using the logit model reported in Table S3. A propensity score 2 is the conditional probability of labor force nonparticipation in year t+1 and is also estimated using the logit model reported in Table S3.

**Figure 6:**
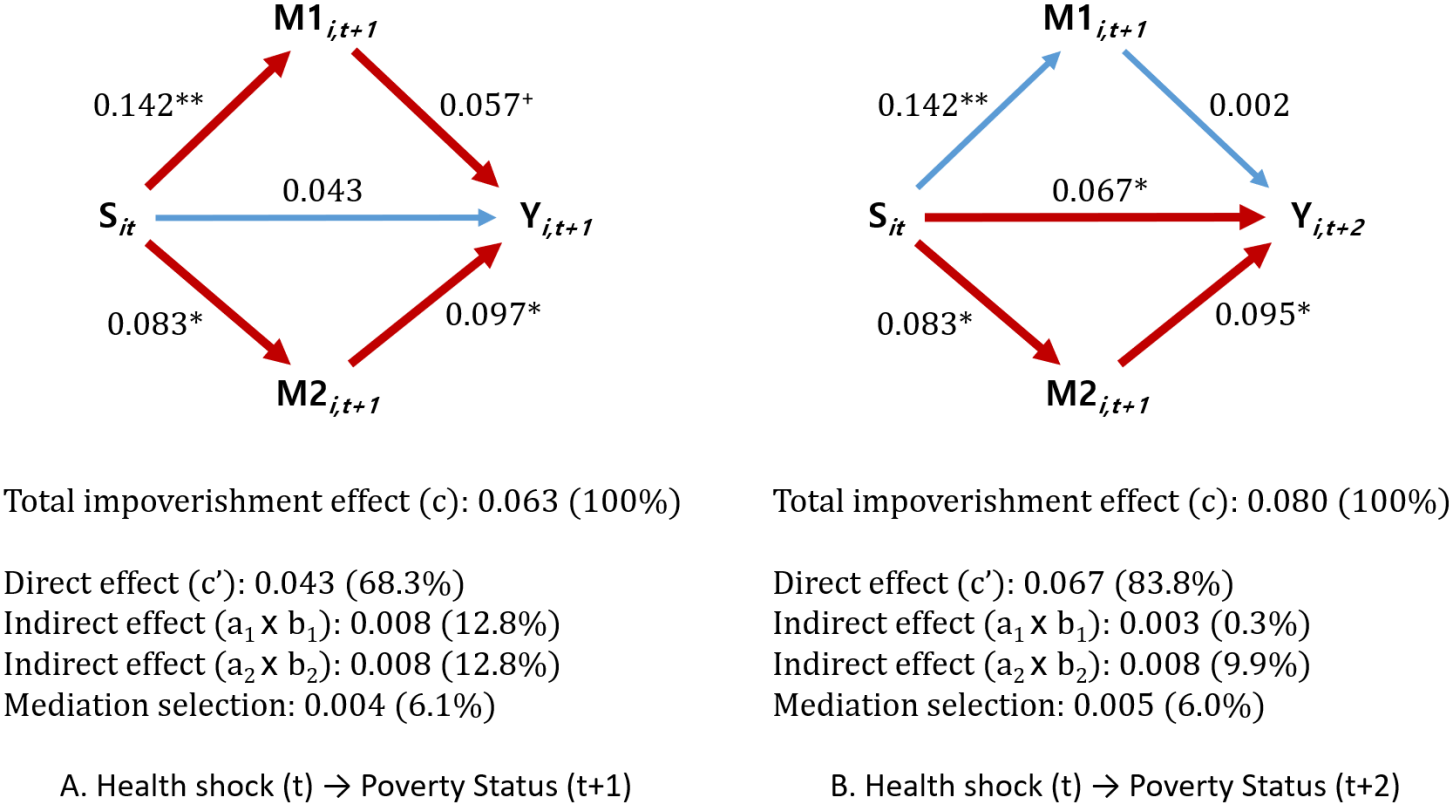
Decomposition Analysis to Explain the Overall Effects of a Health Shock on poverty Status After 1 and 2 Years. (S, health shock, M1, catastrophic medical expense, M2, labor force nonparticipation, Y, poverty status; ^+^*P* < .010, *P < .050, **P < .001). Source: Korean Welfare Panel Study, 2007–2016

Figure 6 shows the result of the decomposition analysis indicating how each mediator contributes to the total effect of medical impoverishment. As shown in Figure 6A, a health shock induces impoverishment after one year through both the medical expense and work capacity pathways, which explain 12.8% [=100 × (0.083 × 0.097)/0.063] and 12.8% [=100 × (0.142 × 0.057)/0.063] of the total effect, respectively. However, when we decompose the mediation effect of a health shock on poverty status after two years (Figure 6B), we find that a health shock leads to poverty mainly through labor force nonparticipation [9.9% = 100 × (0.083 × 0.095)/0.080]. The magnitude of the total indirect effect markedly decreased at year t+2 (10.2% = 0.3% + 9.9%) compared to year t+1 (25.6% = 12.8% +12.8%) because mediator variables were measured at t+1. The direct effect of a health shock on impoverishment was still an important pathway explaining the delayed effect of medical impoverishment.

## Discussion

 Using Korea as an example, this study presents causal estimates of the effects of a health shock on poverty status by using panel data from the KOWEPS. In this study, a health shock increases the absolute poverty rate by 4.6–8.0 percentage points, and the effect is sustained for up to four years after the onset of illness. On average, a sudden hospitalization reduces annual non-medical expenditures and equivalized disposable income by just over 3.2 million KRW (2500 USD) and 1.2 million KRW (1000 USD), respectively.

 The magnitude and duration of the effects of a health shock on household consumption and income were found to be larger than those suggested in previous studies.^6,9,50-53^ For example, in studies conducted in Third World countries, the effects on household consumption were relatively small and often reported to be statistically insignificant.^51,53,69^ In studies conducted in Western countries, the economic effects of a health shock tended to disappear over time.^49,70-72^ Some of those differences could be due to differences in measurement methods of health shock and outcome variables such as indicators (eg, activities of daily living) or threshold (eg, three days).^9,50,51,53^ However, we suspect that they are also due to differences in institutional contexts — such as healthcare systems and labor market characteristics — among countries.

 Impoverishment effects are not the whole story, however. Selection effects are also important.^49^ The current phenomenon of medical impoverishment could reflect two different processes: (1) selection into the health-shock group, and (2) impoverishment caused by the health shock. Households that experienced a health shock were typically more disadvantaged before the onset of illness than households at risk of illness that did not experience a health shock. In this study, the selection effects were 5.6–8.2 percentage points. Thus, both health selection and social causation play a role in explaining the process of medical impoverishment, and we can say that there is a poverty → health shock → poverty pathway in Korea, which supports the existence of a medical poverty trap.^1^ Policy alternatives should therefore be enacted in both directions to alleviate health inequalities.

 In this study we hypothesized that medical impoverishment would arise through two separate mechanisms: (1) medical expenses and (2) work capacity. Our analysis showed that hypothesis to be partially true. The mediation effects we found operate in more complicated ways than we expected. A health shock induced temporary poverty (ie, through year t+1) through both the medical expense and work capacity pathways. However, chronic poverty (ie, after year t+2) appears to be mediated only through labor force nonparticipation. This information provides important suggestions for policy alternatives. An employment protection scheme to prevent labor market exit after the onset of a severe illness must be considered more important than a cash transfer program to reduce the out-of-pocket burden.^44,73^ In addition, a sickness benefit should be designed in conjunction with other kinds of income security schemes (eg, disability insurance, unemployment insurance, public assistance) to protect workers’ households for a longer period. According to the results of this study, labor force nonparticipation due to a health shock tends to last for at least four years after the onset of an illness.

 This study contributes to current knowledge and provides several lessons. We provide a simple, easy-to-use measurement indicator for a health shock. Until now, health shocks have been defined in various ways,^6,7,9,10,19-30,37,51-54,57-60,69-72^ and that lack of consensus was an important barrier to researchers wanting to conduct studies of medical impoverishment. In this study, we defined a health shock as volatility in the number of inpatient days. Days of hospitalization is a general indicator already collected in many panel studies.

 Despite our useful outcomes, this study has some methodological limitations. First, the size of the study sample was relatively small. Although we began with ten years of KOWEPS panel data for 13 670 households, only 398 of those households were found to have experienced a health shock as we defined it. Although the 796 households used in the analysis are sufficient for statistical verification, the probability of false negatives can increase when the sample size is small. Indeed, in this study, the impoverishment caused by a health shock in some of the analytic models had a significance level between 0.05 and 0.10. Second, since we assembled the analytic data by pooling all health shock and 1:1 matched cases that occurred over the three consecutive years, duplicate sampling was found in 18 out of a total of 798 households. Specifically, 10 households were assigned to the treatment group in a specific year but were resampled to the control group in another year, and the other 8 cases were assigned to the control group in two different years (ie, non-exclusive sample size of 780). These methodological limitations were reviewed through sensitivity analysis that artificially excluded duplicate cases before using the matching method (Table 2-D). Third, the choice of analytic method could be problematic. In this study, we verified differences in the outcome variables between the health-shock and non–health shock groups using a chi-square or nonparametric test (Kruskal-Wallis test) at each time point. Autocorrelations among repeatedly measured variables were not seriously considered. In this case, the standard error of the estimated values would generally underestimated and the t-scores overestimated, so that a type 1 error becomes possible.

 In conclusion, the present results indicate that a health shock, when no household members were hospitalized in the previous year but they together experienced more than 30 days of hospitalization in this year, is a cause of poverty. The association between a newly developed illness and impoverishment was strong, and a temporal pattern was evident in the causal mediation analysis. Logical plausibility was relatively high because our results are consonant with previous studies concerning the medical poverty trap. Thus, our findings provide additional evidence for recommending an employment protection scheme to prevent labor market exit after the onset of a severe illness. In addition, an income stabilizing scheme, such as a sickness benefit after health shock, should be introduced as a policy alternative in Korea to protect people from medical impoverishment.

## Acknowledgements

 I am grateful to Inhoe Ku, Baekeui Hong, Taejin Lee, Yoon Kim, and Ji-young Bae for their helpful comments on an earlier version of this paper. I also thank to anonymous reviewers who provided valuable comments on the manuscript.

## Ethical issues

 Ethical approvals were obtained from the Institutional Review Board of Seoul National University (E1708/001-001).

## Competing interests

 Author declares that he has no competing interests.

## Author’s contribution

 COK is the single author of the paper.

## Supplementary files

Supplementary file 1 contains Tables S1-S3.
