# Supplementary material for: Effect of Health Shocks on Poverty Status in South Korea: Exploring the Mechanism of Medical Impoverishment
Source: Int J Health Policy Manag. 2021 Sep 5;11(10):2090–102. doi: 10.34172/ijhpm.2021.97 (PMC9808294; doi:10.34172/ijhpm.2021.97)
Supplement: Supplementary file 1 — contains Tables S1-S3. [file ijhpm-11-2090-s001.pdf]

Article title: Effect of Health Shocks on Poverty Status in South Korea: Exploring the Mechanism of Medical Impoverishment

Journal name: International Journal of Health Policy and Management (IJHPM)

Authors' information: Chang-O Kim[1,2]

[1]Visiting Doctors Program of Medical Home, Seoul, Republic of Korea.

[2]Institute of Social Welfare, SungKongHoe University, Seoul, Republic of Korea.

(*Corresponding author: nation@skhu.ac.kr)

Supplementary file 1

Table S1. Composition of the study sample.

Year	07	08	09	10	11	12	13	14	15	16
Entire sample of KOWEPS	6314	6207	6034	5735	7532	7312	7048	6914	6723	6581
Study sample										
Health shock occurrence (t=2010)				*						
Balanced panel 1	4813	4813	4813	4813	4813	4813	4813	4813		
Subsample a (health shock group)	128	128	128	128	128	128	128	128		
Subsample b (non-health shock group)	4685	4685	4685	4685	4685	4685	4685	4685		
Health shock occurrence (t=2011)					*					
Balanced panel 2		4553	4553	4553	4553	4553	4553	4553	4553	
Subsample a (health shock group)		153	153	153	153	153	153	153	153	
Subsample b (non-health shock group)		4400	4400	4400	4400	4400	4400	4400	4400	
Health shock occurrence (t=2012)						*				
Balanced panel 3			4304	4304	4304	4304	4304	4304	4304	4304
Subsample a (health shock group)			117	117	117	117	117	117	117	117
Subsample b (non-health shock group)			4187	4187	4187	4187	4187	4187	4187	4187

Notes: The study sample consisted of three balanced panel samples missing no data for the major variables for eight years, from year t-3 to year t+4, based on the point at which a health shock occurred (t). * Indicates the baseline year when a health shock occurred within the study sample.

Table S2. Logit analysis to estimate the conditional probability that a health shock occurred in 2010, 2011 or 2012 (N=13,670).

	Balanced panel 1<br>OR (95% CI)	Balanced panel 2<br>OR (95% CI)	Balanced panel 3<br>OR (95% CI)
Conditional variables			
Inpatient days (t-3)	1.00 (0.99-1.01)	1.00 (1.00-1.01)	1.01 (1.00-1.01)*
Subjective health (t-3)	1.06 (0.82-1.37)	0.94 (0.75-1.18)	0.80 (0.61-1.05)
Number of households of chronic illness (t-3)	1.33 (1.02-1.74)*	1.12 (0.89-1.40)	1.03 (0.80-1.32)
Number of households of disability (t-3)	1.36 (0.99-1.89)	0.94 (0.67-1.33)	0.69 (0.45-1.06)
Number of households of smoking (t-3)	1.32 (0.90-1.96)	0.72 (0.49-1.06)	1.46 (0.97-2.19)
Number of households of alcohol abuse (t-3)	0.84 (0.49-1.42)	1.61 (1.04-2.50)*	1.14 (0.68-1.89)
Forgone medical care (t-3)	1.35 (0.57-3.24)	0.45 (0.11-1.85)	2.19 (0.84-5.73)
Disposable income poverty (t-3)	0.88 (0.40-1.90)	0.80 (0.41-1.54)	2.28 (0.90-5.76)
Discretionary income poverty (t-3)	1.36 (0.63-3.64)	1.19 (0.61-2.32)	0.55 (0.21-1.40)
Absolute poverty (t-3)	0.60 (0.35-1.05)	1.18 (0.70-2.00)	1.11 (0.59-2.09)
Labor income (t-3)	1.00 (1.00-1.00)	1.00 (1.00-1.00)	1.00 (1.00-1.00)
Transfer income (t-3)	1.00 (1.00-1.00)	1.00 (1.00-1.00)	1.00 (1.00-1.00)*
Private medical insurance (t-3)	1.00 (1.00-1.00)	1.00 (1.00-1.00)	1.00 (1.00-1.00)
Financial asset (t-3)	1.00 (1.00-1.00)	1.00 (1.00-1.00)	1.00 (1.00-1.00)
Financial debt (t-3)	1.00 (1.00-1.00)	1.00 (1.00-1.00)	1.00 (1.00-1.00)
Disposable income poverty (t-2)	1.52 (0.63-3.64)	0.78 (0.39-1.55)	1.91 (0.66-5.51)
Discretionary income poverty (t-2)	0.44 (0.18-1.05)	1.32 (0.67-2.60)	0.56 (0.20-1.59)
Absolute poverty (t-2)	1.23 (0.69-2.21)	1.05 (0.63-1.75)	0.77 (0.39-1.51)
Labor income (t-2)	1.00 (1.00-1.00)	1.00 (1.00-1.00)	1.00 (1.00-1.00)
Transfer income (t-2)	1.00 (1.00-1.00)	1.00 (1.00-1.00)	1.00 (1.00-1.00)
Private medical insurance (t-2)	1.00 (1.00-1.00)	1.00 (1.00-1.00)	1.00 (1.00-1.00)
Financial asset (t-2)	1.00 (1.00-1.00)	1.00 (1.00-1.00)	1.00 (1.00-1.00)
Financial debt (t-2)	1.00 (1.00-1.00)	1.00 (1.00-1.00)	1.00 (1.00-1.00)
Number of family members (t)	1.03 (0.78-1.36)	1.00 (1.00-1.00)	1.00 (1.00-1.00)
Age of householder (t)	0.95 (0.83-1.08)	0.97 (0.86-1.08)	1.19 (1.02-1.39)*
Age^2 of householder (t)	1.00 (1.00-1.00)	1.00 (1.00-1.00)	1.00 (1.00-1.00)
Living with children under 20 (t)	0.52 (0.24-1.13)	0.50 (0.25-0.99)*	1.47 (0.70-3.09)
Living with parents over 65 (t)	1.51 (0.84-2.71)	1.42 (0.84-2.41)	0.89 (0.49-1.63)
Female householder (t)	0.69 (0.38-1.26	1.10 (0.60-2.03)	0.98 (0.47-2.04)
Householder's educational year 10-11 (t)	2.04 (1.13-3.67)*	1.01 (0.63-1.61)	0.93 (0.56-1.54)
Householder's educational year ≥12 (t)	0.68 (0.29-1.62)	0.48 (0.25-0.93)*	0.29 (0.13-0.64)*
Divorced or separated householder (t)	1.37 (0.74-2.54)	0.88 (0.47-1.66)	0.69 (0.33-1.45)
Unmarried householder (t)	0.71 (1.84-2.75)	0.85 (0.29-2.50)	1.36 (0.36-5.10)
Living in urban area (t)	0.95 (0.64-1.40)	0.94 (0.65-1.36)	0.78 (0.52-1.17)
Beneficiary of medical aid (t)	1.02 (0.14-7.60)	3.01(0.01-1611)	1.00 (1.00-1.00)
Arrearage of health insurance premium (t)	1.27 (0.18-9.06)	0.58 (0.00-308)	1.00 (1.00-1.00)
Model fitness test			
c-statistics	0.746	0.672	0.729
Hosmer-Lemeshow test: χ^2-statistics (*p*-value)	5634.46 (<.001)	4362.67 (.948)	4028.3 (.996)

Abbreviations: OR, odds ratio; CI, confidence interval

Notes: Balanced panels 1, 2, and 3 report results using the subset of households at risk of a health shock in 2010 (n=4813), 2011 (n=4533), and 2012 (n=4304), respectively. Coefficients with * have a p-value below the significance level of 0.05.

Discrimination and calibration ability were tested using c-statistics and the Hosmer-Lemeshow test, respectively.

Table S3. Logit analysis to estimate the conditional probability of a catastrophic medical expense and labor force nonparticipation measured in year t+1 (n=796).

	Catastrophic medical expense (t+1) OR (95% CI)	Labor force nonparticipation (t+1) OR (95% CI)
Conditional variables		
Catastrophic medical expense (t-3)	1.38 (0.96-1.97)	
Catastrophic medical expense (t-2)	1.42 (0.98-2.06)	
Catastrophic medical expense (t-1)	1.91 (1.30-2.81)*	
Labor force nonparticipation (t-3)		2.23 (1.25-3.98)*
Labor force nonparticipation (t-3)		1.81 (0.93-3.52)
Labor force nonparticipation (t-3)		8.53 (4.60-15.81)*
Number of family members (t)	0.67 (0.53-0.83)*	0.45 (0.33-0.61)*
Age of householder (t)	1.12 (0.99-1.27)	0.84 (0.72-0.99)*
Age^2 of householder (t)	1.00 (1.00-1.00)	1.00 (1.00-1.00)*
Female householder (t)	1.20 (0.72-2.00)	2.37 (1.24-4.53)*
Householder's educational year 10-11 (t)	0.94 (0.60-1.48)	1.15 (0.63-2.10)
Householder's educational year ≥12 (t)	1.75 (0.86-3.54)	0.73 (0.25-2.19)
Divorced or separated householder (t)	0.49 (0.28-0.85)*	0.47 (0.23-0.97)*
Unmarried householder (t)	0.67 (0.21-2.14)	0.35 (0.07-1.67)
Living in urban area (t)	0.80 (0.57-1.11)	1.69 (1.03-2.76*
Model fitness test		
c-statistics	0.727	0.923
Hosmer-Lemeshow test: χ^2-statistics (*p*-value)	673.81 (.484)	758.7 (<.001)

Abbreviations: OR, odds ratio; CI, confidence interval

Notes: 796 propensity score–matched samples were used for analysis. Coefficients with * have a p-value below the significance level of 0.05. Discrimination and calibration ability were tested using c-statistics and the Hosmer-Lemeshow test, respectively.
